# Digital twin-enabled interactive cockpits for smart products management and testing

**DOI:** 10.3389/frai.2025.1685702

**Published:** 2025-10-17

**Authors:** Matti Rachamim, Jacob Hornik

**Affiliations:** ^1^Matti Rachamim, Graduate School of Business Administration, Bar-Ilan University, Ramat-Gan, Israel; ^2^Jacob Hornik, Coller School of Management, Tel-Aviv University, Tel-Aviv, Israel

**Keywords:** digital twins, AI, human-in-the-loop (HITL), product management, technology adoption model (tam)

## Abstract

Digitalization is influencing the design, development, and management of products across myriad industries, transforming traditional products into smart ones. Among digital technologies and models, the digital twin (DT) is regarded as an important contribution to the advancement of physical entity management. DTs are virtual representations of physical objects or systems, which are continuously updated with real-time data collected from their physical counterparts. Surprisingly, DT has yet to be applied in marketing. This study aims, accordingly, first, to introduce the DT concept and, second, to explore the human factor (human-in-the-loop) in DT. Third, elaborate on the DT cockpit (the DT’s interactive element) in the product management paradigm. Specifically, the authors use vehicles as a case study to show how interactive digital twins (IDTs) can be employed to predict and optimize vehicle performance, reliability, sustainability, and customer satisfaction. To conceptualize IDT for smart products and marketing analytics, the customer-centric Technology Acceptance Model (TAM) is employed. As this is the first study to explore DT technology in marketing, the DT concept’s main attributes are discussed, significant contributions are suggested, and avenues for future research are delineated.

## Introduction

*“Volvo, the renowned automobile company for ensuring the best passenger safety uses digital twins. They create virtual replicas to test and try out different materials and aerodynamics of new vehicle designs as well as in-vehicle communication components. This way, they can choose the ideal design that would improve performance, create fuel-efficient models, and enhance passenger* satisfaction” (Blake et al., 2024, p. 44).

Intense competition, rapid technological development, and constantly changing consumer preferences are forcing marketing to be more efficient and agile in delivering products and value to customers. As a result, marketing is increasingly turning towards new-age technologies, such as artificial intelligence (AI), the Internet of Things (IoT), big data, blockchain, cloud and fog computing, mobile internet, drones, etc., to design smarter products and enhance interaction with stakeholders (Gnizy, 2024; Kumar and Kotler). The digital revolution, furthermore, has precipitated a sweeping shift from traditional product design and manufacturing to a smart product approach in which existing equipment, processes, software, and devices are retrofitted with smart sensors and other cyber-physical systems (CPS) (Kannan and Li, 2017; Paul et al., 2024). With the prominence of personalization and customer engagement as go-to customer management strategies, marketers need to understand how to integrate the latest technological advances into their existing practices to seamlessly generate actionable insights. Developments such as eased networking, declining computing prices, nanocomponents, and accretive device connectivity have enabled companies to seamlessly integrate and virtually replicate various tangible and intangible entities (Tao et al., 2018; Tao and Qi, 2019; Timperi et al., 2023). The term ‘digital twin’ (DT) has been coined to refer to this type of modeling. By owning and controlling information of affiliated entities, DTs improve planning, management, and forecasting (Bala et al., 2024; Reim et al., 2023). Despite the considerable volume of recent research dedicated to DT implementation in business process management (Ivanov, 2024; Korepin et al., 2024), the phenomenon remains woefully underexplored in marketing. The present study, therefore, seeks to introduce the marketing community to DT as a groundbreaking technology that promises to advance interactive marketing.

While AI, big data analytics, and the marketing Internet of Things (MIoT; Kumar and Kotler, 2024) have paved the way for the emergence and use of DTs as means of ‘twinning’ the lives of physical entities in a range of fields (Stacchio et al., 2022), the advent of eXtended Reality (XR) in industrial and consumer electronics has introduced novel paradigms that may be used to visualize and interact with DTs. Indeed, XR technologies that support human-to-human interactions for training and remote assistance could transform DTs into collaborative intelligence tools (Wang et al., 2024b) that will enable human-machine interaction by voice, gesture, motion, touch, etc. Furthermore, as all these ‘smart devices’ and ‘smart things’ are connected, overviews can be aggregated into DTs (Kobayashi and Alam, 2024). Thus, a major issue in smart marketing concerns how emerging technologies can be integrated (Gnizy, 2024; Kannan and Li, 2017) for unified decision-making and predictive maintenance (Monek and Fischer, 2025; Paul et al., 2024). DT technology undoubtedly will play a central role in addressing such problems (Attaran et al., 2024).

The widespread digitization of products is creating vast digital traces of functions and services, which can be transformed into valuable data. This data supports intelligent decision-making and cost-effective business solutions, particularly in fast-moving industries such as automotive (Kaiser et al., 2019). As vehicles increasingly become electric, digitized, interconnected, and intelligent (Wadhwa and Babbar, 2023), it is essential to adopt a human-centered approach that connects drivers, vehicles, and infrastructures. This approach must also account for non-driving activities in automated vehicles (AVs). A comprehensive strategy that integrates emerging technology-based solutions, facilitated by advancements in sensor technology and data science, appears promising (Gupta et al., 2020; Varadarajan et al., 2010). Given the need for highly automated vehicles to accommodate a range of technical and manual functions, these systems will demand unprecedented flexibility in the human-vehicle interface.

One way to address this issue is by using an interactive digital twin (ITD), which can monitor and simulate all human-vehicular interactions and communications. An umbrella term for IDT is DT cockpit (Bana et al., 2022), which provides a graphical user interface for visualization of data organized in digital shadows (Romero et al., 2020) and models, and for interaction with DT services. Thus enabling stakeholders to access, adapt, and add information, as well as monitor and partially control the physical product. Since smart products generate vast quantities of data, reducing such data to an amount the DT can process is crucial. Thus, the digital shadow contains precisely the data the DT requires to perform its task (Romero et al., 2020). Moreover, shadows may contain information from different perspectives, e.g., systems (physical and organizations), processes, products, and humans (Davila-Gonzalez and Martin, 2024).

## Smart marketing and smart products

Smart marketing is considered an important evolution, which is expected to drastically alter how consumers engage with marketing as we know it. For clarification, the term ‘smart’ in this context represents all things embedded in or enhanced by technology. Accordingly, whenever data are collected from different sensors, actuators, and machines within a marketing environment and access to and control of the data and the devices generating it are enabled through the internet, smart marketing is in play, and such a scenario may be termed a ‘marketing Internet of Things’ (MIoT; Kumar and Kotler, 2024). The MIoT in this sense will focus mainly on the transfer and control of mission-critical information and responses and rely heavily on machine-to-machine communications. Recent developments in smart marketing include AI language models such as ChatGPT, Google Gemini, and Meta Llama, which can provide a vast array of new marketing data, as well as novel ways in which people interact with computers and each other. Modern smart marketing research frequently utilizes big data, derived from a vast number of observations across various subjects, brand SKUs, predictor variables, and periods. This data fills extensive databases, producing large volumes of diverse information. For instance, Amazon and AliExpress collect data on millions of product units, along with detailed demographic information. Similarly, retailers have access to extensive datasets, thanks to the deployment of RFID (Radio Frequency Identification) devices, product reviews, social networking sites, mobile marketing, e-commerce platforms, and customer requirements (Ivanov, 2024).

Smart products (or ‘product avatars’) also comprise cyber-physical systems, which contain semi-autonomous functions and can communicate with other products or other ecosystem components via internet-based services (Paul et al., 2024). They differ from conventional products in their capabilities, which include intelligence, autonomy, and connectivity. An intelligent product possesses a unique computer-readable identifier, monitors its status and environment, stores data about itself, shares and receives information, and is capable of decision-making. Its intelligence comes from an embedded or remote computer with network access (Barricelli and Fogli, 2024).

## Study objectives

Given the dearth of DT use in marketing hitherto, in the present study, we advocate for the future deployment of IDT in smart marketing ecosystems. Inspired by recent successful DT applications, such as the digital twinning of Paris 2024 Olympic venues (Medium, 2024), which enable stakeholders to negotiate the uncertainty and difficulties inherent in organizing large-scale events while promoting sustainability and customer experience (CX), we propose a single DT framework that can synchronize data and communication protocols across multiple devices and stakeholders, to support data exchange and information interaction, between real products and their virtual twins in any scenario, anywhere, and at any time.

Smart vehicles are examined here as a case study to demonstrate how IDT can be employed to predict and optimize product performance, reliability, sustainability, and thus customer satisfaction. Furthermore, we show how a complete IDT framework allows end-users to simulate future events, capturing interactions between consumers, the environment, and products, thereby enabling a better understanding of operational risks and the remaining useful life of products. While the automotive sector is used here as an illustrative case, the IDT cockpit framework is conceived as scalable and adaptable across industries with varying levels of technological complexity. In lower-complexity domains, such as smart home thermostats, lighting systems, or wearable devices, the cockpit can function as a streamlined interface, requiring less computational intensity while still delivering value through predictive maintenance, energy efficiency, and personalized user comfort. By tailoring the scope of integration to the specific industry context, the IDT cockpit demonstrates both cost-effectiveness and adaptability, ensuring that its core principles remain valid across a wide spectrum of smart products. We follow the general frameworks and propositional inventories delineating a conceptual entity in marketing research (MacInnis, 2011). Thus, our study can be seen as one of envisioning, as MacInnis (2011) terms it, in that it seeks to call our attention to “what we have been missing and why it is important,” and “reveal what new questions can be addressed” (p. 138).

To advance these objectives, precisely during an era in which marketing scholars are calling for more conceptual work (e.g., Deighton et al., 2021; Vargo and Koskela-Huotari, 2020), we first provide an overview of DT, IDT, and related concepts relevant to marketing management. Second, we outline a conceptual framework for IDT-enabled smart product management using, for illustration purposes, the Technology Acceptance Model (TAM; Gonzalez, 2024; Lala, 2014). Third, we elaborate on theoretical and significant marketing applications. Fourth and finally, we identify several fundamental research challenges emanating from our conceptualization for the managerial exploitation of IDT in smart product monitoring. The study, we suggest, goes beyond a literature review by offering compelling observations of marketing in the real world. Employing a multi-perspective approach, it aims to deliver valuable insights about IDT, lending perspective on several impacted interactive marketing areas. Specifically, we use motor vehicles as a case study to demonstrate how IDT can be employed to simulate the behavior of physical automobiles, predict and improve their performance, and optimize operation, reliability, sustainability, disposal, recycling, and customer satisfaction, ultimately leading to a more efficient and innovative automotive industry.

Among product management models, our conceptualization incorporates the consumer-centric TAM (Gonzalez, 2024) into the widely embraced five-dimensional DT model framework (5DT, Attaran et al., 2024, Ivanov, 2024, Korepin et al., 2024), enhanced by stakeholder intuition (human-in-the-loop, HITL; Retzlaff et al., 2024). This conceptualization, we maintain, can help marketing to take full advantage of the theoretical and practical benefits of the prescribed approach, while also highlighting important avenues for future research geared toward enhancing effective decision-making. Each of these endeavors requires additional details, which, due to considerations of readability and space constraints, we furnish in an extensive Web Appendix.

## DT fundamentals

*“Digital twin is revolutionizing industries”* (Ma et al., 2024, p. 102).

The National Academies of Sciences, Engineering, and Medicine (NASEM) recently published a report titled *Foundational Research Gaps and Future Directions for Digital Twins* (National Academies of Sciences, Engineering, and Medicine, 2024, pp. 147–49), which underscores the scientific contributions and significance of Digital Twins (DTs). This report identifies key areas where DT technology can advance research and industry practices, emphasizing its role in enhancing the integration of digital and physical spaces, especially with the rise of the metaverse. Digital Twins (DTs) are cloud-based systems that integrate data from various smart, data-generating resources. In the context of smart products, DTs collect extensive product-related information, revolutionizing data integration and system modeling across industries such as healthcare, housing, manufacturing, and energy (Holmes et al., 2021). This technology allows for the creation, use, management, and maintenance of virtual counterparts (twins) of physical entities or systems, facilitating real-time, two-way data exchanges (Bala et al., 2024). Through the continuous collection of data via IoT sensing devices, DTs can dynamically simulate real objects and environments, constructing high-fidelity virtual models that accurately mirror their physical counterparts (Kobayashi and Alam, 2024; Wang and Wang, 2019).

**Figure 1:**
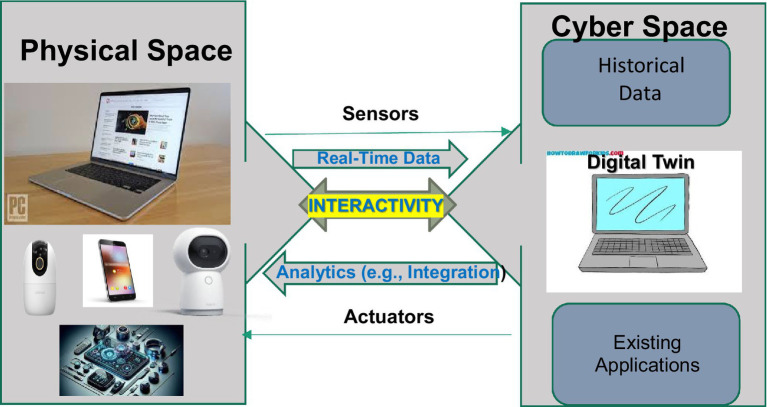
IDT visual illustration.

Interactive Digital Twins (IDTs) are designed to significantly enhance human-machine interactions and inter-machine communications by dynamically emulating their real-world counterparts. These systems integrate both raw and processed data to reflect actual conditions accurately. With advanced simulation models, IDTs can monitor and control increasingly complex systems. Furthermore, IDTs are equipped with basic analytical models and software that allow for rigorous analysis and prediction of system conditions under various scenarios. At their most advanced level, IDTs incorporate machine learning models that generate real-time insights, enabling immediate optimization of system performance (Turner et al., 2016). Table 1 summarizes the key characteristics of DTs, while Figure 1 visually illustrates the concept of IDTs. Additional elaborations can be found in Appendix 1.

**Table 1:** Major IDT characteristics.

Characteristics	Description
State	The value of all parameters of both the physical and virtual twin in their environment
Physical process	The process in the real-system environment that will change or impact the state of the physical twin
Virtual process	The process in the virtual environment (e.g., research) that will change or impact the state of the virtual twin
Virtual environment	The technology-based environment in which the virtual twin exists
Physical entity (twin)	The real entity (e.g., products, consumers, firms, devices)
Virtuality	The virtual digital twin synchronized with the physical entity at a twinning rate
Synchronization and integration (twinning)	Real-time integration and convergence of physical systems and their digital counterparts
Twinning rate	The rate or frequency at which synchronization occurs
Networking devices	Physical or cloud-based communication devices for data exchange
Cloud computing	The delivery of computing services, including servers, storage, databases, networking, software, research, and intelligence, over the internet (“the cloud”) to offer faster innovation, flexible resources, and economies of scale
Data storage	Acquiring historic data of an entity for data comprehension
Heterogeneous data	Ability to handle large amounts of data from different sources and formats
Self-adjustment	Self-adaptation and parameterization capabilities following changes in the system during its lifecycle
Information selection	Identifying, extracting, and storing useful information
Pattern identification	Identifying changes and trends via data analysis
Physical-to-virtual connection	Data transfer from the physical entity to the virtual environment
Close-loop feedback	Feedback is provided to the systems and other digital twins, using interfaces to assess the computing information
Metrology	Measuring the current state of the physical/virtual entity
Optimization	Achieving best outcomes while addressing data uncertainty
Simulation	Representing current status and what-if scenarios
Location	Enables users not co-located to collaborate in design and implementation

What makes DT unique is the convergence of virtually all the latest cutting-edge technologies, including big data, the Internet of Things (IoT), social networks (SN), virtual reality (VR), augmented reality (AR), haptic interaction technology, voice interaction technology, gesture recognition technology (Kobayashi and Alam, 2024), as well as the more recently developed DT cockpit (Bana et al., 2022), driven by major advances in cognitive science, machine learning and AI. IDT integrates all the processes by which sensory input is transformed, elaborated, reduced, stored, recovered, and used.

Based on insights and simulations, DTs can recommend changes to their physical counterparts. Likewise, IDTs are dynamic models that serve as real-time symbiotic “virtual replicas” of real-world systems. Inter alia, they can leverage Dynamic Data Driven Applications Systems (DDDAS) bidirectional symbiotic sensing feedback loops to provide continuous updates. In this context, reconfiguration decisions may be autonomous or interactive. For example, HITL-DDDAS systems generate considerable amounts of data useful for myriad applications, such as improving performance, predictive maintenance, training, etc. Consequently, IDT will be able to steer simulation, measurement, analysis, management, and reconfiguration aimed at more accurate modeling and analysis. DT’s value spans a multitude of domains. For example, Korepin et al. (2024) have shown empirically that DTs tend to enhance companies’ operational efficiency, as evidenced by a high correlation between DT expenses and company revenues.

In sum, IDTs are virtual representations or models of physical objects, systems, or processes, which are designed to be interactive, allowing users to engage with them in real-time and manipulate various parameters or elements to simulate different scenarios, analyze performance, or test important issues. IDTs are essentially DTs with an added layer of engagement. The interactive part takes this a step further. It allows users to not just observe the DT, but also interact with it while learning and adapting from its outcomes and recommendations.

### Adaptive learning

Adaptive learning (Reim et al., 2023) is a crucial aspect of any DT approach. It refers to DTs’ ability to continuously learn from new data and update their models to improve accuracy and effectiveness over time. This process allows the system to adapt to changes detect emerging patterns and make it more robust against ever-evolving new technologies.

## Human digital twin (HDT)

*“Digital Twin is at the forefront of the Industry 4.0 revolution facilitated through advanced data analytics and the Internet of Things (IoT) connectivity”* (Sharma et al., 2022, p. 101).

The term human digital twin (HDT), which extends the DT concept, has been applied recently in numerous domains, including medicine, manufacturing (Wang et al., 2024b), and sports performance (Barricelli and Fogli, 2024). A digital human can be defined as a life-like being, powered by artificial AI, with the capability of conversing, interacting, and creating an emotional connection, like any other human being. HDTs have the potential to change the practice of human system integration as they employ real-time sensing and feedback to tightly couple measurements of human performance, behavior, and environmental influences throughout the life cycle. In recent years, a growing number of studies have borne witness to the fusion of human factors with advanced digital technologies such as the Internet of Things (IoT), artificial intelligence (AI), and eXtended reality (XR). For instance, unobtrusive, body-worn sensors, embedded in, among other things, inertial measurement units (IMUs) and wireless wearable electromyography (EMG) devices (Davila-Gonzalez and Martin, 2024), are utilized for on-site measurement, enabling biomechanical analysis during work. These innovations are invaluable as they facilitate the provision of accurate data for virtual-real mapping of humans throughout production or service stages (Wang et al., 2024a).

Similar to DT, HDT is presented in the literature as a replica, copy, or counterpart in cyberspace, or the digital world, of a real person in the physical world (Davila-Gonzalez and Martin, 2024; Wang et al., 2024a). The HDT concept has been proposed as a critical method for realizing human-centricity in an array of smart applications (Ma et al., 2024). HDTs are also distinguished from animated characters by one key characteristic, i.e., “the illusion that they are ‘just living life’ like the rest of us” (Bala et al., 2024, p. 340). Emotion AI (Petrescu and Krishen, 2023), also known as *artificial emotional intelligence*, refers to machines’ ability to measure, understand, simulate, and react to human emotions. Recently, AI researchers have made significant technical advancements, developing machines that are increasingly able to detect users’ emotions and adapt their responses. HDTs are considered powerful tools for designing personalized services and optimizing satisfaction and lifestyle. However, surprisingly, they have been almost wholly overlooked in marketing and consumer research.

## Conceptual framework

**Figure 2:**
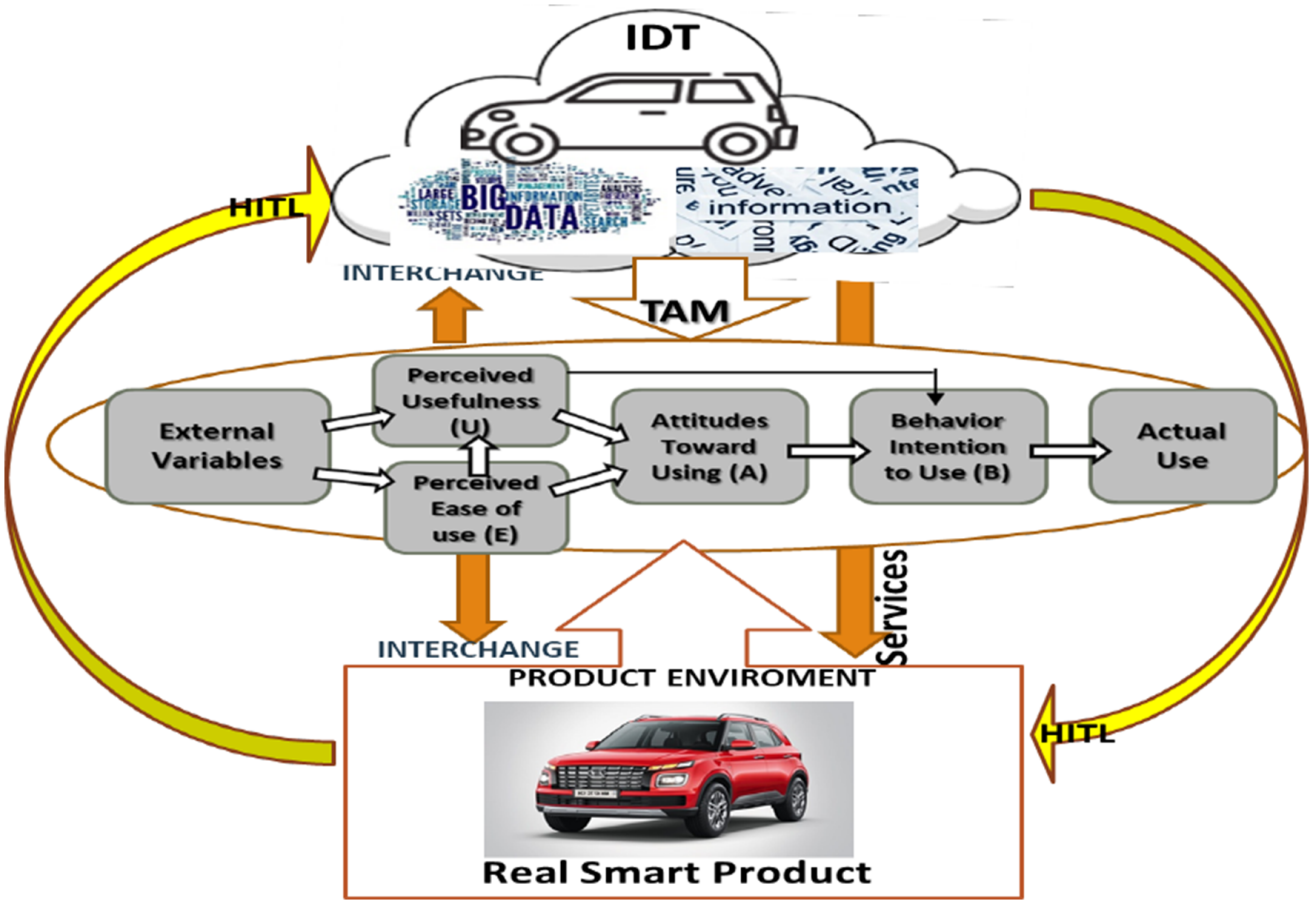
Digital twin-enabled technology acceptance model (TAM) of a smart product.

Our novel conceptualization of an IDT-enabled TAM for smart products is depicted in Figure 2. To advance conceptualization, we have adopted the commonly used and validated five-dimensional DT model framework (5D-DT, Ma et al., 2024; Tao et al., 2018) along with the HITL concept, thereby integrating the most advanced data-generating smart technologies, devices, and human intuition, to support TAM of a smart product. In this case, a motor vehicle. We propose a novel IDT framework that can synchronize the data and communication protocol across multiple devices to support data exchange between the real product and virtual product in any scenario, anywhere, and at any time. Our framework can support the synchronization of many different sensors and actuators. Furthermore, we show how a complete IDT framework allows end-users to simulate future events capturing the interactions between the environment, consumers, and products, enabling a better understanding of operational risks and the remaining useful life of assets.

While TAM and the five-dimensional DT model may appear to emphasize distinct domains, user perceptions of technology adoption on the one hand, and technical system architecture on the other, their integration is inherently complementary. TAM contributes the consumer-centric perspective, ensuring that smart product frameworks address perceived usefulness and ease of use, thereby supporting user acceptance. In parallel, the five-dimensional DT model provides the architectural and operational foundations required for system stability, real-time synchronization, and data-driven decision-making. By combining these two perspectives, the framework achieves a comprehensive balance between human adoption and technical robustness, bridging subjective perceptions with objective technological design.

### Technology acceptance model (TAM)

The Technology Acceptance Model (TAM), one of the most influential theories explaining technology acceptance, posits that two primary factors tend to influence consumers’ intention to use a new smart product: (1) perceived usefulness, and (2) perceived ease of use (Gonzalez, 2024). Indeed, TAM has proven to be a parsimonious model that explains much of the variance in users’ behavioral intention related to smart technology adoption and usage across a wide variety of contexts. Thus, TAM has been validated as a reliable theoretical model for exploring consumer acceptance of smart products and services, from smart watches to smart vehicles to smart homes to smart sharing (Yoon and Cho, 2016). Indeed, it has been widely used hitherto by scholars to study the online service context, and in particular users’ behavioral patterns and willingness to pay, enjoying popularity in marketing specifically as a consumer-centric model (Ritz et al., 2019). Accordingly, TAM may be considered uniquely suitable to the proposed framework.

## The five-dimensional DT/IDT model

Among the various DT classifications, the five-dimensional model is the most commonly used (Attaran et al., 2024). It posits that DT architectures are comprised of five key components, the first of which is the *physical entity*, i.e., the real-world object (smart product) or system on which the DT is based. It can be anything from a simple machine to a complex infrastructure, such as a power grid. Sensors installed on the physical entity gather data about its operation, performance, and environment.

The second dimension is the *digital model*, the virtual counterpart of the physical entity. It is built using data collected from sensors and may include 3D models, mathematical equations, and software simulations. The digital model captures the essential characteristics and behavior of the physical entity in a virtual environment.

The third dimension is *data connection*, the vital link that ensures two-way interaction between the physical entity and the digital model. Real-time data from sensors flows into the digital model, keeping it updated on the physical entity’s current state. The digital model can also send commands back to the physical entity, thus influencing its operation in real-time.

The fourth dimension of the IDT model is *services and analytics*. Here data and insights from the digital model are used to furnish valuable services. These can include performance monitoring, anomaly detection, predictive maintenance, recommendations, and even optimization of the physical entity’s operation.

The fifth and final dimension is *real-time feedback and optimization*, which closes the loop, allowing the digital model to directly influence the physical entity.

## IDT models and supporting technologies

The three major pillars of any Digital Twin (DT) are models, software, and supporting technologies. The primary advantage of an IDT lies in its ability to incorporate all design models according to predefined rules and a recommender system. Several model types are essential for enabling IDTs, each contributing unique strengths and fulfilling specific purposes. For example, the SMARTBUY geo-marketing model utilizes Wi-Fi access points (APs) installed on a store’s premises to detect customer proximity (Bourg et al., 2019). Six key models are crucial for the functionality of IDTs: data models, physical models, machine learning models, behavioral models, functional models, and virtual sensors models (Sharma et al., 2022; Stacchio et al., 2022). Each of these models is reviewed in detail in Appendix 2.

### The IDT cockpit

The cockpit is the user interaction part of an IDT, it supplies the graphical user interface (GUI) for visualizing data ORGANIZED in digital shadows, modeling, and interaction with IDT services (Kobayashi and Alam, 2024). Interactive technology refers to “methods, tools, or devices that allow various entities to engage in mediated communication to facilitate the planning and consummation of interactions between them” (Varadarajan et al., 2010, p. 97). A cockpit can be seen as both a special service furnished by the IDT and an integrative front-end component for various specific services that the IDT offers. In short, the IDT cockpit provides a central hub for all the data and functionality associated with the IDT. Thus, IDTs highlight the bidirectional interaction that comprises feedback flows of information from the physical system to the virtual representation to update the latter, and from the virtual back to the physical system to enable decision-making, either automatically or with HITL. In other words, the virtual-to-physical interaction is the process that results in the transfer of information from the virtual representation back to the physical entity (Bana et al., 2022). This interaction closes the IDT loop by allowing the insights and decisions generated through the virtual representation to be realized in the physical system, either through actions that result in a change in the physical system or those used to collect additional information from the physical system to further update the virtual representation. The term cockpit has been adopted from the airline industry (Dalibor et al., 2020). Think of it like this: The physical airplane is the real world, the IDT is a complex computer model of the airplane, and the IDT cockpit is the flight deck where pilots can see all the information gathered from the models. By the IDT cockpit, ‘pilots’ can gain four key benefits. The first is improved monitoring: the condition of the airplane can be tracked in real-time to identify potential issues before they become problems. The second is enhanced decision-making: data collected from the IDT can be used to make better decisions about how to operate and maintain the airplane. The third is reduced costs: by identifying and fixing problems early on, money can be saved on maintenance and repairs. The fourth is increased safety: IDT cockpits can help to improve safety by allowing operators to identify and mitigate potential risks. In sum, the cockpit is an information processing device that facilitates interaction between all items and stakeholders based on advances in human-machine interfaces. Thus, by using digital and other technologies, IDTs and stakeholders have myriad ways of interacting.

#### Human-computer interaction (HCI)

Human-computer interaction (HCI) is a dynamic approach that goes beyond traditional marketing to create a two-way conversation between DTs and consumers (Wu and Huang, 2023). It focuses on engaging consumers and encouraging active participation through interactive elements and personalized experiences. Like any user-computer interface, it employs specific methods of interaction with the consumer. Progressively, over the years, there has been a shift from the “Hands & Touch” era, in which human-machine interaction was done manually through buttons, keyboards, and switches, to the “Mind & Body” era, in which, to support the flow of information being used as a user interface, the human body is utilized, for example, through visual or auditory messages (Diao et al., 2023). Voice user interfaces (VUI) are another technology that facilitates human-computer interaction. Effective VUIs allow users to request information through natural language without learning a specific query syntax. For well over a decade now, consumers have experienced VUIs through digital assistant (DA) technologies such as Siri, Alexa, and Google Assistant. Key aspects of human-computer interaction include the following (Barricelli and Fogli, 2024; Wang et al., 2024b):

*Engagement:* consumers actively interact with the IDTs, rather than passively receiving marketing messages. This can involve liking, sharing, commenting, or participating in discussions on social media, providing feedback, or actively seeking information.

*Two-way communication:* interactive consumer behavior involves a dialogue between consumers and IDTs. This can take place through comments, reviews, live chats, or forums where consumers can ask questions and receive responses in real-time. *Co-creation:* Consumers may actively participate in the creation or customization of IDT. For example, crowdsourcing ideas, voting on product features, or submitting user-generated content.

*Feedback loop:* HCI enables IDTs to gather feedback directly from consumers, allowing them to better understand consumer preferences, needs, and pain points. This feedback loop can inform IDT smart product development, marketing strategies, and customer service improvements.

*Real-time interaction:* IDT thrives on real-time interaction. This means that IDTs are responsive to customer inquiries and feedback and adapt their monitoring systems based on customers’ engagement data.

*Focus on consumer-generated content:* IDT encourages consumers to create and share content, including reviews, photos, videos, social media posts, etc. User-generated IDT can be a powerful tool for building trust and credibility among customers.

*Cross-user interaction:* IDTs hold considerable potential for cross-company (user) interaction. Indeed, shared IDTs can extend application boundaries to cross-marketing and enable data exchange between multiple stakeholders. Far from being limited to internal applications, IDTs represent a suitable instrument for cross-marketing collaboration.

The cockpit IDT layer also provides a hub for machine-to-machine (or system-to-system) interaction (Paul et al., 2024). Data interoperability is fundamental in the context of any DT because it allows effective data sharing, unlocking barriers to interactivity and understanding. Like DT, IDT is capable of optimizing the broader system beyond its boundaries by exchanging information with other, interconnected IDTs, thus allowing decisions to be taken jointly with the respective IDTs in the interconnected systems, leading to enhanced performance through joint optimization. Implementing IDT interoperability requires realizing data integration and data exchange (Bala et al., 2024).

### IDT data fusion

*“Digital Twins thrive on data integration”* (Tao and Qi, 2019, p. 490).

Like all DTs, IDTs can integrate diverse technologies (de Koning et al., 2023), models, and data from heterogeneous sources. For example, they can gather data from the Internet of Things, blockchain, AI, and supplier collaboration portals (Ivanov, 2024), to accurately simulate and assess smart product design decisions (Qi et al., 2021). One of the major pitfalls in marketing and consumer research arises from the daunting task of integrating and synchronizing the vast array of consumer and product traces obtained from autonomous obtrusive and non-obtrusive data-generating devices (Bala et al., 2024). Thus, data integration is a crucial need in marketing research with multiple analytical implications in terms of conceptualization, illustration, convergent validation (triangulation), development of analytic density, and decision-making. Clearly, complex, diverse, and heterogeneous data may hinder marketing research, which relies on data gathered from smart sensors and other data-generating sources. This complexity elevates integration and interoperability challenges on both a syntactical and semantic level.

#### Simulation

IDTs for smart products are designed, among other capabilities, to simulate different smart product scenarios (Hutabarat et al., 2016). Understanding the fundamental differences between a typical simulation and an IDT is critical to the success of any IDT application. The former is an offline conditional experimentation (Ma et al., 2024), whereas the latter is a real-time event in which the quality of the IDT model determines how accurate any simulation will be. IDT technology explores how the users’ interaction is captured by MIoT sensors and actuators, while the loss of information between the real and simulated smart product is kept vanishingly small. With the aid of AR, discrete products’ events can be overlaid with simulation model layouts in real-time over the real product via smart devices. Case studies involving virtual reality (VR) representations of marketing settings boosted by motion and depth sensors, such as Kinect, might yield promising results (Turner et al., 2016). IDT models can be constructed using real-product layouts for managerial control of discrete event simulation capturing real-time entity operation and voice commands (Wang et al., 2024b). In the case of vehicles, radio frequency identification (RFID) technologies may be used to monitor and manage facilities and services, focusing on the visualization of logistics trajectories (Zhong et al., 2016).

Real-time synchronization of heterogeneous data streams presents significant challenges, particularly in terms of latency, protocol compatibility, and system stability. To address these issues, IDT cockpits can leverage fog and edge computing architectures to enable local data processing, thereby reducing transmission delays. Adaptive buffering strategies further support resilience against fluctuating data loads, while standardized communication protocols such as MQTT or OPC-UA enhance interoperability across diverse devices. In complex environments such as congested urban traffic, system robustness is reinforced by prioritizing safety-critical data streams and employing redundancy mechanisms to maintain reliability. Together, these measures ensure that IDT cockpits achieve both timeliness and stability in real-world scenarios.

#### Human-in-the-loop (HITL)

Modeling and analysis of systems equipped with sensors and connected to the internet are becoming more automated and less human-dependent. However, bringing expert knowledge into the loop along with data obtained from Internet of Things (IoT) devices minimizes the risk of making poor and inexplicable decisions, and helps to assess the impact of different strategies before applying them in reality. While IDTs are more of a data-driven simulation of the physical smart product, IDTs can bring a human dimension into the modeling and simulation. IDTs demonstrate a close association with human-computer interaction (HCI) and human-machine interaction (HMI), both of which focus on establishing seamless interfaces between humans and IDTs (Barricelli and Fogli, 2024).

The ‘human-in-the-loop’ (HITL) concept is also known as interactive analytics, in which analytic algorithms occasionally consult human experts for feedback and course correction (Retzlaff et al., 2024). In such cases, it becomes crucial to integrate human supervision, along with expert knowledge, experience, and justifications, into an IDT. This integration aims to enhance comprehension of the unknowns within (cyber)physical systems and to refine the design of the underlying data-driven methodology. The accuracy and reliability of IDT models depend heavily on the quality of the data they are fed. The consumer/human-in-the-loop approach is a unique ingredient that can ensure the success of these technologies.

Human intuition within IDT systems can be reflected through measurable proxies, such as gaze direction, voice tone, or physiological indicators, which are captured through sensors and translated into structured data streams. These signals provide real-time cues of user perception and situational awareness, enriching the simulation environment. When conflicts arise between algorithmic outputs and human inputs, for example, in emergency braking scenarios, arbitration is handled through a layered mechanism. In safety-critical cases, human intervention is prioritized to ensure trust and accountability. In less critical situations, algorithmic decision-making prevails, supported by adaptive learning that incorporates past interactions to refine system responses. This balanced approach preserves both human oversight and technical robustness.

As advanced technologies revolutionize TAM monitoring, HITL might be a crucial element for comprehensive and effective IDT oversight. This approach is often used in situations in which AI systems are unable to make decisions or perform tasks autonomously due to complexity, uncertainty, or ethical considerations, which are typical in the automotive industry (Kaiser et al., 2018). HITL sources might encompass all relevant stakeholders, including experts, informed managers, employees, and most importantly, customers (Wu et al., 2022). Current visual IDT allows for a vast upgrading of interactional capabilities, steering expert judgment through visually presented aspects of data characteristics (Diao et al., 2023).

HITL systems offer several benefits. First, they enhance accuracy and performance by enabling stakeholders to provide feedback and corrections to AI systems. Second, they can render IDT systems more transparent and explainable, which in turn can help to build trust and confidence in their use. Third, they can help ensure that product IDTs are used safely and ethically. Fourth, they allow stakeholders to stay informed about changes in regulatory requirements and make certain that the smart product complies with the latest standards. Fifth, they permit stakeholder involvement in monitoring and controlling the IDT. Sixth, HITL systems can help provide labels and annotations to unsupervised learning data, thus improving the accuracy of IDT models.

In complex smart products, human expertise is often necessary for handling intricate situations, making critical decisions, and adapting strategies based on contextual factors not fully captured by automated systems (Diao et al., 2023). For example, self-driving cars can use HITL in a machine/car-learning approach to ensure the safety of passengers and pedestrians. While the vehicle’s sensors detect obstacles, human drivers can provide additional feedback to ensure it make accurate real-time decisions. Finally, stakeholders can improve the IDT by providing information that is difficult to obtain via smart technologies (Murali et al., 2022).

#### Recommendation models

A DT recommendation engine (RE) is a type of software that leverages the concept of IDT and recommendation algorithms to provide personalized recommendations or insights. Recommendation lists may include products, services, offers, vendor-web sites, etc. REs use algorithms that consider consumer data such as current position, purchase history, shopping lists, and browsing behavior (e.g., use of keywords for product searches or website views). In this context, curation tools, capable of searching large databases and creating recommendation shortlists, have become popular because they can save time, elevate brand visibility, and increase connection to customers. The techniques used in recommendation systems generally fall into three categories: (1) content-based filtering, which uses a single customer’s data, (2) collaborative filtering, the most prominent approach, which derives suggestions from many other customers, and (3) knowledge-based systems, which are based on specific customer queries, and generally employed in complex domains, where the first two techniques cannot be applied. This approach can be hybrid, for instance, where content-based filtering exploits individual metadata and collaborative filtering finds overlaps between customer preferences. Such systems build a profile of what a customer buys and then look at what other customers with similar profiles purchase. Content summarization is another fundamental tool that can support recommendation services (de Koning et al., 2023). Machine learning (ML) approaches have been developed as well to perform content-based recommendations. For a detailed review of deep learning for recommendation systems (see Batmaz et al., 2019).

#### Task-specific models

Task-specific models (TSMs; Shi et al., 2023) refer to ML models that are designed and optimized for specific tasks or types of tasks. They are trained on data that are relevant to the particular task they are meant to perform, which allows them to achieve high performance and efficiency concerning the task in question. TSMs allow the IDT system to understand what steps need to be carried out, in what order, and under what conditions. They can refer to different activities such as decision-making, problem-solving, learning, and perception (Bala et al., 2024). Modern AI models can learn from millions of examples to help find new solutions to difficult problems. However, building new systems tends to take time and a large amount of data. The next evolution in AI will involve a shift from task-specific models to *foundation models*, large-scale models trained on massive sets of unlabeled data that can be adapted for various tasks with minimal fine-tuning (Yang et al., 2023). These advancements in AI, particularly the development of foundation models, align closely with the evolving landscape of IDT models and their supporting technologies. Together, they will create a fertile and cutting-edge domain for the advancement of smart product management and customer experience (CX) research.

## IDTs for smart cars

*“Cars are becoming computers on wheels”* (Murali et al., 2022, p. 211).

Driving is a social activity that involves endless interactions with other entities on the road. In recent years, the automotive industry has faced disruptive changes. Inter alia, it finds itself undergoing a revolutionary shift from offering goods and related services to offering data-supported services that meet customers’ needs. The transition toward e-mobility, autonomous driving, and ubiquitous connectivity will offer new value to stakeholders (Blake et al., 2024). Naturally, as in any other computerized system, autopilot or self-driving capabilities in motor vehicles are achieved through the integration of hardware and software components. The hardware comprises a suite of sensors and cameras, while the software employs sophisticated algorithms to create a neural network for data processing and decision-making. This process simulates the human brain and operates in a more precise and efficient manner. The focus of attention and available sensory modality of the driver, i.e., the most appropriate sensory channel for efficient interaction, are estimated based on the monitoring activities that are constantly running in the background. Among automakers, Tesla and Volvo reportedly are integrating DT technologies into every car it produce. The partner company that developed Tesla’s DT application, Thinkwik, has asserted that real-time mechanical issues in Tesla motors, regardless of their magnitude, are being fixed by simply downloading over-the-air (OTA) software updates (Moiz and Alalfi, 2023).

In theory, any car can be digitally ‘twinned’, that is, everything in the vehicle itself can have an IDT to which it is linked. Digital trace data encompasses a wide range of information, including web browsing history, location data, social media activity, communication data (e.g., emails), online purchases, app usage, device information, sensor data, network activity, and cognitive advanced driver assistance systems (ADAS) data (Diao et al., 2023). In the automotive industry, DTs are equivalent to a high-fidelity, virtual blueprint of the entire car and its performance, down to the smallest part. They are a dynamic tool that reflects every part of a vehicle in real-time, going beyond traditional modeling to yield insights unthinkable hitherto. Indeed, by creating virtual replicas of vehicles, stakeholders (manufacturers, retailers, service providers, managers, and customers) can experience a host of advantages previously beyond reach. Vehicle IDTs, for instance, allow for analysis of individual driving habits, thus optimizing vehicle performance based on actual usage patterns, with external source data (weather, traffic, etc.), visual analytics, automatic speech recognition (ASR), and ADAS software for detecting possible future threats all coming into play. Ultimately, IDTs will have the capacity to integrate all relevant consumer data, including personality traits and physiological data, and convert it into business value, while supporting various stakeholders’ decisions. Other notable technologies include radar and LiDAR (light detection and ranging) systems, as well as road operator cameras for managing traffic flow. General Motors (GM) has created digital twins to collect data about their equipment’s performance and predict maintenance issues. As a result, they can proactively tackle these issues, thus increasing the equipment’s lifespan (Moiz and Alalfi, 2023).

Consumers are increasingly demanding vehicles that are intelligent and user-friendly, that is, smart cars. Automotive smartification involves equipping vehicles with photographic lenses, laser radar, and other sensing apparatuses, which are coupled with operating systems and AI chips to achieve data access, interconnection, and automated driving. Thus, embedded in the smart vehicle’s ‘cockpit’ is an elaborate sensing and monitoring system, which acts as its ‘five senses,’ incorporating in-vehicle, smartphone and individual user device sensors (e.g., pulse transmitter belts). The development of smart cockpits thereby expedites the shift from a vehicle-centric to a consumer-centric model. Moreover, by improving chip performance to endow products with new functions, the driver-vehicle relationship can be radically redefined in conformance with the “decoupled-but-collaborative software and hardware” model (Moiz and Alalfi, 2023). Indeed, in line with evolving industry demands, the smart cockpit system promises to transform the vehicle from an ordinary transportation tool into an ecosystem that integrates travel, life, and customers’ delight. The domain of interaction in particular involves the monitoring of sensation, perception, information exchange, inference, and decision-making (Barricelli and Fogli, 2024).

With the help of AI recognition technology, vehicles are invested with the ability to listen, speak, see, and think, just like people. Drivers normally must constantly gather information from various car sensors about their surroundings to make safe driving decisions. However, human drivers are also subject to limited perceptibility and distractions. Failing to know where entities are and predict what they will do is liable to result in serious safety hazards. Traditionally, the responsibility for avoiding such hazards rests solely with the driver. One of the chief advantages of IDT technology in this context lies in the fact can be transferred across different vehicles, bringing to bear interactions between driver, vehicle, and environment. With features such as sophisticated environmental awareness, accurate decision-making logic, and collaborative and comprehensive controller units (Blake et al., 2024), IDT will enhance safe driving. Also, IDT enables driver-vehicle interaction using Conduct-by-Wire vehicle guidance, where the primary driving tasks (braking, accelerating, and steering) are assigned to the vehicle (Wadhwa and Babbar, 2023), and the driver’s input is automatically converted into a movement vector, with the primary driving tasks being performed without further driver assistance (Diao et al., 2023). IDTs that use the driver as a sensor will enable overtaking maneuvers when the sensors are blocked or suggest maneuvers to the vehicle.

Likewise, when an IDT interface has learned a specific driver’s preferences, it can enable similar functions in different vehicles. Thus, when the driver changes vehicle, the system can update the new vehicle using the most up-to-date personalized settings, regardless of the different interior layout. What will be transferred is not necessarily the layout of an icon, but the logic of how information should flow across the different sensory channels and displays (Chen et al., 2018). This clearly would have beneficial effects in terms of standardization and adoption of safety criteria for automated vehicle interfaces as well.

Furthermore, IDT interactive vehicle-to-vehicle (V2V; system-to-systems interaction) technology allows profiles to be shared among neighboring vehicles and used to estimate the potential risks of collision depending on the actions taken by the drivers. Indeed, the IDT interface enables a high level of driver and passenger connectivity, which is particularly relevant for safety-oriented applications stemming from V2V or vehicle-to-infrastructure communication, in which the risks entailed in available actions are visualized to drivers so they can take appropriate action to avoid collision.

Voice-user interfaces (VUIs) are another advanced HCI technology of vital importance for smart car IDT, as they allow voice commands to be used to safely perform specific vehicle functions. Taking advantage of voice recognition and synthesized voice response, VUIs historically have opened up new horizons and opportunities for both conventional users and those with disabilities, thus making great strides in digital accessibility. In the automotive market, they are becoming an integral part of standard equipment, capable of recognizing drivers’ voices to enable them to safely access a range of services, often custom-tailored to the needs of each driver and with ample opportunity for customization. Adoption is facilitated by the increasing prevalence of connectivity that links more and more devices into daily experience, from the home to the car. This is a growing trend also due to the increasing pervasiveness of smart vehicles. Today it is possible to take Siri, Google Assistant or Alexa with us in the car (Diao et al., 2023).

A vehicle’s behavior is determined by its driving context, which includes road conditions, nearby elements, infrastructures (e.g., traffic lights), and even drivers’ mental states. With better sensors and connected technologies, vehicles’ capacity to read the driving context is improving. IDT technology offers context-based interpretations of data gathered from drivers, their vehicles, and the environment, as well as the interactions between them. For each entity, information is stored and updated over time, to allow driver reactions to be assessed about previous states and environmental conditions. The data obtained subsequently can be translated into a meaningful *percept* of the overall state, which in turn can be shared with other road users. Vehicle IDT will play a fundamental role in understanding and shaping the interactional dynamics between humans and smart vehicles. With increasing levels of automation, drivers will have more time and choice to perform various tasks other than driving, and this opens up new avenues for interaction (Diao et al., 2023).

### Multimodal interaction

Multimodal systems in user interaction are defined by Murali et al. (2022) as “those that process two or more combined user input modes, such as speech, pen, touch, manual gestures, gaze, and head and body movements, in a coordinated manner with multimedia system output” (p. 201). As with mono-modal interaction, multimodal interaction can have multiple inputs and outputs, offering drivers different methods of interacting with vehicles depending on the driving situation and the driver’s cognitive state. Furthermore, the drawbacks of any single modality can be compensated for using another modality. One modality may even correct or verify the outputs of another one. Multimodal inputs can be used for controlling vehicle functions in addition to selecting a particular task or object. For instance, a mixture of three modalities, voice, gaze, and movements, can be used to pick vehicle objects, such as side mirrors or windows, and then control these objects with gestures or speech (Blake et al., 2024).

### Implicit versus explicit interactions

Typically, users can interact with an intelligent vehicle implicitly as well as explicitly (Diao et al., 2023). Implicit interactions are able to estimate and infer driver action states such as fatigue or drowsiness, cognitive state, emotions, and even posture or pose recognition, which can convey certain cues to the intelligent vehicle. The user might not be consciously aware that their actions are being interpreted as inputs. In explicit interaction, by contrast, these actions are clear, deliberate, and usually involve clear input devices or commands. The user communicates with the vehicle or vice versa using deliberate button clicks, voice commands, gestures, and communication through haptic and display interfaces. The user is consciously aware of the interaction. Explicit interactions are intentional, while implicit interactions are inferred from actions that might not be intended as direct communication with the IDT system. Explicit interactions are usually visible and clear to the user, while implicit interactions are often subtle or hidden. Understanding the distinction between these types of interactions is crucial for designing smart products that can effectively and intuitively respond to user needs (see Appendix 3 for more on this subject).

#### Self-driving/autonomous vehicles (AV)

Self-driving/autonomous vehicles (AVs) of the future promise to change the face of transportation as we know it, and by extension our very lives. AVs exchange information with both other vehicles (V2V) and the infrastructure (V2I), while smartphone sensors and individual user device sensors (e.g., pulse transmitter belts) can boost the amount of available vehicle data. A typical smartphone, for example, contains an acceleration sensor, an ambient light sensor, a temperature sensor, a barometric sensor, a gyroscope sensor, a fingerprint sensor, a magnetic field sensor, and so on. Communication interfaces commonly found on smartphones include Wi-Fi, GPS, near-field communications (NFC), Bluetooth, and infrared (IR) LED, while numerous additional sensors are available for physiological measurements (Qi et al., 2021). However, in view of the fact that self-driving vehicles need to interact and communicate with their surroundings, including people, vehicles, and roads, to efficiently operate, it is evident that interactive intelligence is also of vital importance. Thus, as self-driving capabilities advance, the focus on designing human-AV interfaces that support interactive systems will surely intensify. A vehicle that will be able to effectively and safely realize unmanned driving will need to interact not only with the passengers inside the vehicle, but also with pedestrians, other cars, and road conditions outside of the car. As the external environment is subject to a vast array of variables such as distance, noise, temperature, humidity, wind speed, etc., it is clear that the IDT smart car’s decisions and judgments will be determined by multiple factors, thus posing one of many challenges for the car industry. Regardless, the driver’s role will shift gradually from one of active control of the vehicle to one of supervision and intervention when necessary. Lastly, integrating blockchain technology into the IDT will help to secure vehicle data management and communication. For example, a DT of a vehicle connected to a blockchain network can be used to store and manage vehicle data securely, thus allowing automobile experts to track a vehicle’s performance and maintenance history in real-time.

In sum, more and more automakers are investing in research and development for human-vehicle interaction, to attract and satisfy customers. As such interaction becomes more humanized, vehicles will become more intelligent, convenient, and appealing. IDT provides a unique opportunity for drivers not only to interact with vehicles but also to interconnect with different vehicle components and to benefit from V2V. The advent of autonomous driving technology will fundamentally transform how consumers interact with their vehicles. As IDT technology is further integrated into smart vehicles, profound changes undoubtedly will occur in driving behavior and human-vehicle interaction. The eventual mainstream adoption of IDT-enabled smart vehicles will not only transform automobiles from ‘vehicle-centric’ means of transportation into ‘people-centric’ mobile spaces but also create a new ecology for cars and transportation services.

Dynamic adaptability is embedded in the IDT cockpit architecture through modular and evolutionary design. As new sensors, infrastructures, or communication technologies emerge, they can be integrated into the system via plug-and-play interfaces without the need to reconstruct the entire framework. Ontology-based mapping ensures that new data sources are semantically aligned with existing components, allowing the cockpit to maintain consistency and stability. This modular adaptability enables the IDT model to evolve continuously in response to technological and environmental advances, ensuring that the framework remains both flexible and future-proof.

## Discussion

Digitalization has greatly simplified data collection and analysis methods, which used to be too complex and/or only available to experts. DT is one of the leading data-directed decision-making concepts allowing businesses and manufacturers to simulate products to enhance their speed, cost-effectiveness, and quality. Our study shows that the proposed IDT-TAM framework can improve human-smart product interaction and management requiring less specialist knowledge from stakeholders, and that IDT technologies offer a direct and intuitive method to users concerned with interactive and operational decision support. At the same time, integrating human expertise (HIDT) into the digital domain will significantly bolster IDT’s predictive analytics capabilities.

Digitalization has considerably simplified data collection and analysis, processes that were previously complex and largely limited to experts. Digital Twin (DT) technology is now recognized as a significant data-driven approach, allowing businesses and manufacturers to simulate products to improve product management and quality. Our study suggests that the proposed IDT-TAM framework can enhance human-smart product interaction and management, potentially reducing the need for specialized knowledge among stakeholders. Thus, IDT technologies may offer a more direct and intuitive method for users engaged in interactive and operational decision support. In this conceptual study, we have examined the current state of human-vehicle IDTs, with a focus on automotive sensing technologies and the methods for facilitating natural and intuitive interactions. The importance of interactive interfaces and the need for their development have been outlined. Additionally, we have reviewed state-of-the-art technologies that enable vehicles to understand implicit contextual cues and explicit interaction modes, such as speech and gesture recognition.

IDT technology uniquely focuses on bilateral interdependency between the physical twin and its virtual counterpart. This offers various inherent benefits, as the physical smart product can modify its real-time behavior concurrently in response to the feedback generated by the IDT. Conversely, it allows the simulation to precisely mirror the real-world condition of the physical product. As a consequence, for the car industry, IDT represents a holistic approach that aims to address the main human factors and challenges of smart vehicles. Specifically, an interface based on this approach surrounds the driver and continuously adapts to support any change in their psychophysical state. IDT is meant to increase situational awareness, minimize the obtrusiveness of traditional visual and auditory interfaces, and preserve the driver’s cognitive spare capacity for a prompt and smooth transition of control while providing a comfortable and safe experience. In sum, IDT is a holistic interface designed to mediate the interactions between the driver and the vehicle IDT or any other connected smart entity, as well as between the vehicle and other stakeholders in the external environment (e.g., insurance agents).

### Theoretical applications

*“Digital twins are not just a fleeting trend but an essential component of sustainable innovation”* (Stacchio et al., 2022, p. 498).

IDT development and implementation demands collaboration across multiple disciplines, including logistics, marketing, consumer behavior, data science, computer science, design, and domain-specific fields. Such interdisciplinary collaboration can foster innovation and knowledge exchange. IDT can accommodate existing smart product concepts such as life-cycle management, and TAM. Digital representations of physical products are expected to rapidly transform smart products. Their theoretical implications are vast and still being explored. IDT challenges our traditional understanding of the relationship between physical and digital worlds, but also potentially blurs the lines between the real and the simulated, raising questions about the nature of reality and representation. For instance, what does it mean for a product to be ‘real’ if it has a perfect digital counterpart? (Wang et al., 2024b). By designing and creating a dynamic copy of a smart product, IDT can introduce new properties that do not exist in the latter. This raises theoretical questions about their ontological status and implications for understanding the smart product world.

As this technology continues to develop, we can expect even more profound theoretical questions to emerge about the understanding of AI and virtual space and the role of the different stakeholders in it. As Götz et al. (2020) suggest, significant potential lies in a holistic approach to future digitization initiatives with blockchain-based IDTs, and in positioning the concept as a strategic, multifunctional tool for field support applications. Significantly, recent advances in fog computing in marketing (FC; Hornik et al., 2023; Kumar and Kotler, 2024) promise to shift IDT processing power and data storage away from centralized servers and into local networks where MIoT devices and other monitors are located. Also, with the advent of extended reality (XR; Stacchio et al., 2022), an umbrella term encompassing various immersive technologies that blend physical and digital worlds, including virtual reality (VR), augmented reality (AR), and mixed reality (MR), new theoretical issues might emerge. For example, XR is enabling the creation of hybrid environments where real and virtual products coexist and interact with each other. An MR application, for instance, might allow consumers to place virtual smart products in their actual home or office to see how they might perform before purchasing them. Such emerging technologies will provide users with interactive experiences by integrating digital content into their real-world environments or by immersing them entirely in a simulated way, thus raising new theoretical questions.

### Managerial applications

The introduction of IDTs will allow managers to transition to a predictive maintenance model, which can strike a balance between corrective and preventive maintenance. These efficiencies will enable faster time-to-market with better quality assurance. IDT devices allow outliers, defects, errors, and unexpected consumer behavior to be readily detected. For example, by monitoring and analyzing fuel consumption in various conditions and driving styles, IDTs can suggest optimizations, leading to significant fuel savings over time. Likewise, ensuring compliance with various regulations and standards will be simplified through IDTs, which maintain records and provide a transparent audit trail throughout the various stages of the vehicle’s lifecycle, from conception and design to manufacturing and distribution to use and eventual disposal. As such, automotive IDTs will serve as the central hub of vehicular information, which combines and updates data continuously from a wide range of sources, as a fore noted, as well as from consumer interactivity. Overall, IDTs offer a continuous feedback loop throughout product management, enabling data-driven decision-making, optimization, and innovation. This will lead to improved design, production efficiency, customer safety and experience, and ultimately, sustainability. Thus, IDTs promise to play a major role in automotive TAM by improving predictions and enhancing productivity, profitability, and efficiency. Indeed, by capturing intricate details, analyzing them for deeper insights, and applying knowledge gained from consumers’ interactions, IDTs will offer a roadmap to a future in which motor vehicles are not just smarter but also more resilient, safe, and consumer-centric. The automotive industry can leverage IDTs to provide personalized experiences and services to customers. In other words, by tracing or interacting with customers, individual driving patterns, preferences, and needs can be better understood, thus allowing automakers to tailor recommendations, optimize vehicle settings, and enhance the overall driving experience. Integration of IDT technology enables a holistic understanding of the monitored vehicle, leading to improved decision-making, efficiency gains, and the ability to proactively address challenges in real-time (Bana et al., 2022).

Several emerging technologies are currently being tested that have the potential to significantly enhance automotive IDTs. For instance, voice-based infotainment systems like Apple CarPlay and Android Auto (Moiz and Alalfi, 2023) will allow stakeholders to interact with drivers verbally while collecting cognitive and emotional data during driving. This will lead to a deeper understanding of the driving experience and behavior. Additionally, new algorithms are being developed to estimate and measure mental states and consumer behavior through various indicators, including speech analysis, facial expressions, gestures, posture, movement, and eye tracking, as well as internet and smartphone activity (Bala et al., 2024). IDTs will enable more accurate simulation and integration of these technologies, particularly in the context of autonomous driving. However, it is important to note that most current car IDT systems rely on centralized architectures, which have limitations in ensuring trusted data provenance, secure and tamper-proof data storage, and reliable traceability. To address these challenges, key technologies such as blockchain and especially Fog Computing are being explored as solutions.

### Fog computing-enabled IDTs

Fog computing (FC) is a distributed computing architecture that sits between the cloud and data-generating devices. It works by bringing some of the processing power and storage closer to where the data is created, rather than relying solely on the cloud. FC provides the distributed computing infrastructure and capabilities that enable real-time data processing, reduced latency, improved scalability, enhanced privacy, and local decision-making for IDTs. This will make it a key enabler for realizing the full potential of IDTs in TAM applications (for a recent review, see Hornik et al., 2023; Kumar and Kotler, 2024; and for more on DT applications, see Appendix 4).

### Study limitations

Although the use of theoretical models like TAM is beneficial in developing IDT’s applicability to product management, it is important to highlight the drawbacks. For example, it must be acknowledged that while extensive support can be found for the use of the TAM models, there is paucity in the marketing literature that can furnish a basis for more advanced IDT applications.

The adoption of IDT technology in this context also comes with its own set of challenges. Ensuring data privacy and security is crucial, as IDTs often involve the representation of sensitive personal and financial information. Also, it cannot be ignored that AI-enabled IDT is still in its nascent stage. Overall, many research questions remain unanswered. An acknowledgment of the aforementioned challenges is imperative, toward filling at least some of the gaps in the literature.

## Future research

*“The development, maintenance, and evolution of digital twins are still challenging research areas”* (Bana et al., 2022, p. 69).

Although algorithmic improvement is noteworthy in the case of IDTs, the application of the IDT paradigm in product TAM is a completely new development, which exposes a stark gap in the research literature. Thus, accuracy measures (e.g., mean absolute error, root-mean-square error) should be deployed to check the robustness of the proposed framework (Ma et al., 2024). Notably, as well, the framework presented avoids distinct sub-categories of its dimensions to reduce complexity and leave room for individual focuses on current and future applications. A refinement of the model, consequently, can be part of future work. When using the proposed framework in specific domains with defined modeling techniques and associated tools, specific integration and interaction issues will emerge, thus opening up important research directions concerning data integration, accuracy and reliability, scalability, data privacy and security, and user adoption. Accordingly, among the many relevant research questions that might be posed in future research are the following:

*RQ1*: How would TAM and IDT collaboration resonate with different stakeholders?

*RQ2*: How can the interplay of human (HITL) and machine-generated IDT content be investigated?

*RQ3*: How can marketing research scale decision-makers’ relative trust in different data-generating devices?

*RQ4*: Although IDT has strong data-collection and integration capabilities, very often data contexts are lost, thus creating problems in modeling, especially as concerns emotional data (Diao et al., 2023). How, then, can data loss be prevented/reduced in an IDT for TAM?

*RQ5*: There is a long tradition in marketing and consumer behavior showing different responses to different products (e.g., high/low involvement, hedonic/utilitarian). Do the same differences apply to IDT-based TAM?

*RQ6*: IDT has proven to be an interdisciplinary paradigm. When conducting IDT research, how can marketing benefit from allied disciplines (e.g., economics, computer science, psychology, sociology)?

*RQ7*: How can research integrate useful knowledge extracted from observations of varying natures (traceability information, structural/environment constraints, quality measures) with previous external IDT knowledge to refine a TAM predictive model and enhance or adapt a TAM prescriptive model?

*RQ8*: As the components contained in IDT usually have different properties, the structure of each part and the interaction between different parts tend to be different (Ma et al., 2024). How, then, can ontology-based IDT provide reliable guidance for the implementation of IDT, as well as a way to specify the various components and the relations among them?

*RQ9*: How can human-machine interfaces (HMIs) in smart products foster trust through transparency and explain ability of actions and intentions?

*RQ10*: Being connected to their physical twins, among other things, through (manual) use of recorded data, smart product IDTs generally must deal with missing data. In this case, appropriate techniques for data imputation, which fills in the missing data, should be explored (Ivanov, 2024). Likewise, datasets are liable to contain noisy data points whose distributions are difficult to estimate due to various approaches for constructing the data. What kind of robust algorithms, then, should be developed for such unpredictable noises?

In the future, product IDT research will progress inevitably toward training personnel on new processes, strategies, or equipment within a secure virtual environment (Bala et al., 2024). This will help enhance stakeholders’ skills and reduce the likelihood of errors in real operations. Likewise, it will entail investigating the long-term consequences of IDT implementation, as well as implementation barriers in specific market settings. Finally, conducting case studies across diverse product domains holds significant promise for assessing the practical implementation, advantages, and challenges of ontology-based IDTs, thus yielding valuable insights for real-world adoption. Grappling with these challenges seems well worth the effort given what appears to be IDT technology’s numerous benefits.

## Conclusion

The use of DT technology has been hailed as a groundbreaking development in numerous domains. DT holds immense potential in the metaverse, making it possible to interact with digital versions of people, places, objects, and products of any kind. However, its promise in marketing, smart product management, and research remains far from being realized. By simulating a physical smart product, such as a motor vehicle, in digital form, it can create prototypes with unprecedented accuracy, allowing for analysis and gathering of meaningful feedback before physical production has even begun. If done properly, the value of the enriched data obtained promises to be inherently greater than the sum of any single dataset values combined in the process. Our analysis has shown that HITL-embedded IDTs offer powerful tools for investigating and optimizing automotive TAM. Motor vehicle IDTs, for example, have the potential to transform TAM or any other product model, by providing managers with a new way of understanding and interacting with cars and their customers. By bridging the gap between the physical and digital worlds, managers will be able to improve product research, performance, reliability, sustainability, customer safety, and satisfaction, ultimately leading to more efficient and innovative smart marketing.
