# Supplementary material for: Digital twin-enabled interactive cockpits for smart products management and testing
Source: Front Artif Intell. 2025 Oct 17;8:1685702. doi: 10.3389/frai.2025.1685702 (PMC12586550; doi:10.3389/frai.2025.1685702)
Supplement: Supplementary file 1 [file Data_Sheet_1.docx]

Supplementary Files: **Digital Twin-Enabled Interactive Cockpits for Smart Products**

**Management and Testing**

Sup File 1: More on DT and IDT ………………………………………..………………. 2

Sup File 2: IDT Models and Supporting Technologies 6

Sup File 3: More on Smart Vehicle Interactions 8

WUp File 4: More on Fog Computing for IDT 11

# Sup File 1: More on DT and IDT

A digital twin is a virtual representation of a real-world entity or a system; in other words, a counterpart of a physical object and a process powered by technologies like machine learning (ML), the internet of things (IoT), and analytics. When developing expensive hardware like a cellphone, car, or fighter jet, a virtual model enables virtual collaboration, simulation, or data acquisition for better iterative design. It helps you understand the system’s performance and behavior in a real-world scenario. The virtual replication gives you the answers to the “what if” methods without breaking the prototype. One of the main advantages of a digital twin is that the physical system and its digital twin can co-exist, communicate, and use real-life data from the physical system to improve the simulation. Digital twins are used across industries in several ways. For example, healthcare utilizes this capability for clinical diagnoses, learning, and training, while sectors such as automotive use the ability to optimize the manufacturing value chain, supply chain, product innovation, and enhancements.

Why are digital twins important? Hardware-software partitioning in a parallel design cycle development environment accelerates design schedules. An integrated emulation and prototyping solution can maintain the design integrity that enables iterative and simultaneous verification, which can handle tasks of varied sizes and execution lengths, from smaller IP blocks to subsystems up to the system on a chip (SoC) level. It is also essential to extend this verification effort across the enterprise to other designs being developed concurrently. When managing multiple SoC design projects, it is critical to have a platform that bridges the verification productivity gap to accelerate the verification of SoCs, subsystems, IP blocks, and system-level validation. It is also essential to take a holistic view of the parameters when evaluating emulation throughput. Using a digital twin allows you to verify a chip and the software on that chip. The combined use of the Dynamic Duo emulation and prototyping platforms makes it possible to transition quickly and effortlessly from debugging a chip on the emulation platform to debugging the software running on that chip with the prototyping platform. Digital twins play a crucial role in redefining the emulation architecture with microprocessor-based technology. With digital twin deployments, customers immediately realize improved access to data. As a digital twin matures, other benefits include reduced maintenance costs, more informed process change decisions with large potential savings, and improvements in maintenance and operational efficiency. Having better designs from the start pays dividends over a project’s lifetime, as 80–90% of costs incurred during the production, use, and maintenance of a facility are determined at the design stage. Using digital twins in marketing can improve multiuser collaboration and communication. The ability to interact with data in real-time is changing the way marketing makes decisions. The power to visualize and simulate complex operations in real-time 3D has elevated how people interact with their assets, transforming the way virtually every physical space and asset on the planet is created, built, and operated.

***IDT***

IDT refers to a virtual representation or model of a smart interactions, incorporating various aspects of their physical, biological, and behavioral characteristics. This concept is part of the broader trend of creating cockpit digital twins, which are virtual replicas of real-world entities or systems. The goal of a IDT is to simulate and analyze different facets of a smart product. This can be achieved by collecting and integrating data from various sources, including wearable devices, company records, social media activity, and more.

The IDT concept has potential applications in an array of interactive marketing domains, including healthcare, and overall well-being. By creating an accurate and dynamic digital representation of a smart product, researchers and managers can gain insights into product status, predict potential issues, and tailor interventions or recommendations to specific needs. IDT takes into account real-time data, simulation data, and the fusion of physical and virtual data to meet the needs for personalization and responsiveness. Based on abundant data and models, IDT can construct multi-scale product models to fit different application scenarios and needs for fidelity.

Table 1: **Major IDT Characteristics.**

| **Characteristics** | **Description** |
| --- | --- |
| State | The value of all parameters of both the physical and virtual twin in their environment |
| Physical process | The process in the real-system environment that will change or  impact the state of the physical twin |
| Virtual process | The process in the virtual environment (e.g., research) that will change or impact the state of the virtual twin |
| Virtual environment | The technology-based environment in which the virtual twin exists |
| Physical entity (twin) | The real entity (e.g., products, consumers, firms, devices) |
| Virtuality | The virtual digital twin synchronized with the physical entity at a twinning rate |
| Synchronization & integration (twinning) | Real-time integration and convergence of physical systems and their digital counterparts |
| Twinning rate | The rate or frequency at which synchronization occurs |
| Networking devices | Physical or cloud-based communication devices for data exchange |
| Cloud computing | The delivery of computing services, including servers, storage, databases, networking, software, research, and intelligence—over the internet (“the cloud”) to offer faster innovation, flexible resources, and economies of scale |
| Data storage | Acquiring historic data of an entity for data comprehension |
| Heterogeneous data | Ability to handle large amounts of data from different sources and formats |
| Self-adjustment | Self-adaptation and parameterization capabilities following changes in the system during its lifecycle |
| Information selection | Identifying, extracting, and storing useful information |
| Pattern identification | Identifying changes and trends via data analysis |
| Physical-to-virtual connection | Data transfer from the physical entity to the virtual environment |
| Close-loop feedback | Feedback is provided to the systems and other digital twins, using interfaces to assess the computing information |
| Metrology | Measuring the current state of the physical/virtual entity |
| Optimization | Achieving best outcomes while addressing data uncertainty |
| Simulation | Representing current status and what-if scenarios |
| Location | Enables users not co-located to collaborate in design and implementation |

Sup File 2: **Six Key Models for Digital Twins**

IDTs rely on several key models to function effectively, reflecting various aspects of physical entities in a virtual space. Here are six crucial models for the functionality of IDTs:

*Physical Models*: Are a detailed representation of the physical product, including its geometry, materials, and components. It serves as the foundation for the IDT, providing a virtual counterpart to the physical entity.

*Data Models:* Define the structure and relationships of data collected from the physical product. These models enable efficient data collection, storage, and analysis, supporting decision-making and optimization.

*Behavioral Models:* Simulates the dynamic behavior of the physical product under various conditions. They predict performance, identifies potential issues, and optimizes operations.

*Functional Models:* Represent the functions and processes of the physical product. These models support performance analysis, troubleshooting, and design improvements.

*Virtual Sensor Model:* Create virtual sensors to generate data that might not be available from physical sensors. They Enhance data availability and enables more comprehensive analysis.

*Machine Learning Models:* Utilize algorithms to learn patterns from data and make predictions or decisions. They Enable predictive maintenance, anomaly detection, and optimization.

The specific implementation of these models can vary widely depending on the application. For example, a IDT of a car engine might require highly detailed physical and behavioral models, while a IDT of a building might focus more on energy consumption and occupant behavior models.

**Sup File 3: Implicit/Explicit Vehicle Interactivity**

Recognizing driver and passenger behaviors and activities has far-reaching implications for in-vehicle interaction systems and safety functions. The IDT can be guided to the most appropriate modality (visual/audio/haptic) at each moment if the driver’s intentions are correctly classified. The safety functions, such as airbags, steering, brake, and crash avoidance patterns, can be tailored to the best in-time deployment if the car knows the full body position (sitting, lying, etc.) of its passengers.

Furthermore, with tracking of facial expressions, gestures, and body position, the emotional state and response of the driver and/or passengers can be used to evaluate the automated vehicle’s actions in traffic. Research on safe human-robot interaction and in particular human activity recognition techniques is particularly useful even in the automotive context. Mapping of all passengers in the AV will enable new methods of understanding how not only social interaction between passengers but also between passengers and the intelligent car will look in the future. Upon detecting driver distraction or fatigue, the intelligent vehicle assistant (IVA) may choose to provide visual or vibro-tactile alerts. Furthermore, the IVA can also engage in conversation with the driver to keep them alert. If a nonoptimal body posture is detected, the actuated interiors can nudge the driver to a correct body posture for optimal driving attention. Similarly, if the driver’s mood is found to be angry or sad, the IVA can recommend soothing music, control the in-vehicle temperature, and create a relaxing environment.

Driver Fatigue and Distraction Recognition Driver distraction is one of the major causes of accidents on the road and the US National Highway Traffic Safety Administration (NHTSA) estimates up to 25% of road accidents happen due to some form of driver distraction. In comparison, interior visual sensors such as stereo cameras, IR cameras, ToF sensors, and RGB-D sensors have been widely used to recognize driver and passenger activities, intentions, and behaviors. State-of-the-art neural network architectures for detecting driver distraction such as VGG, AlexNet, GoogleNet, and ResNet have been compared and ResNet architecture seems to outperform other competing strategies. Even single image-based driver activity recognition to detect activities such as talking on the phone, texting, eyes off-road, rubbing eyes, and so on has been demonstrated using neural networks. Emotion recognition is critical for daily function in decision-making, communication, general mood, motivation, and even driving. Emotion recognition is a complex field of research requiring the use of physiological sensors and controlled studies thus increasing the complexity of in-vehicle driver emotion recognition.

In contrast to implicit interaction, users can explicitly interact with the intelligent vehicle assistants by engaging in conversation, proactively performing tasks, gesturing, and so on. The IDT also needs to consider various cultural and geographic aspects while proactively interacting with users. For instance, different gestures can mean different things in Italy or Japan. Furthermore, by analyzing the emotion of the user, the can adapt its interaction mode suitably, thus leading to more empathetic interaction. Explicit interaction can take place via voice-based, display-based, haptic-based, and even multimodal interfaces. Voice-Based Interaction Voice–user interface (VUI) can drastically reduce driver distraction by allowing the driver to interact with the vehicle without taking the eyes off the road or hands-off-the-wheel and reducing the visual cognitive load. Voice assistants are more commonly implemented for OEMs such as BMW Intelligent Personal Assistant with the “Hey, BMW!” prompt, Daimler’s MBUX voice assistant, and also integration of third-party assistants such as Amazon Alexa and Apple CarPlay. The digital voice assistant (VA) needs to understand the commands provided in a naturalistic way without relying on predefined keywords that require prior training for the users. In-vehicle functions such as infotainment, climate control, communication such as making calls or sending texts, vehicle status (such as fuel left) and even assisting with the car by syncing with other voice assistants.

Sup File **4: How can fog computing support IDTs?**

Fog computing (FC), also known as edge computing, involves processing data closer to the source of data generation rather than relying solely on centralized cloud servers. This approach can support IDTs in several ways:

*Reduced Latency:* FC brings processing closer to the edge devices, reducing the latency associated with data transfer to and from distant cloud servers. This is crucial for IDTs, where real-time or near-real-time responses are essential. Faster response times can enhance user experiences in IDTs.

*Improved User Engagement:* IDT often relies on user engagement through various channels, such as mobile apps, websites, or social media. Fog computing enables faster and more responsive interactions, enhancing the overall user experience. This can result in increased engagement with IDT content and activities.

*Data Privacy and Security:* FC can address concerns related to data privacy and security by processing sensitive information locally. This minimizes the need to transmit sensitive data to centralized cloud servers, reducing the risk of data breaches and ensuring compliance with privacy regulations. Enhanced security measures at the edge can protect user data during IDTs activities.

*Offline Capabilities:* FC enables certain processing tasks to be carried out locally, even when the device is offline or has limited connectivity to the internet. This is particularly useful for IDT operations that involve offline user interactions. IDT can collect and process data locally, syncing with the cloud when connectivity is restored.

*Distributed Content Delivery:* FC facilitates distributed content delivery, ensuring that content is delivered efficiently to users based on their location. This is especially beneficial for IDTs that involve location-based targeting, ensuring that users receive content tailored to their geographical context.
